# Evaluation of Human Sperm Quality In Vitro—Purification of Motile Sperm and Subsequent Assessment of Potential Apoptotic Signs Beyond DNA Fragmentation

**DOI:** 10.3390/biom16070928

**Published:** 2026-06-23

**Authors:** Satoru Kaneko, Yukako Kuroda, Yuki Okada

**Affiliations:** 1 Laboratory of Pathology and Development, Institute for Quantitative Biosciences, The University of Tokyo, 1-1-1 Yayoi, Bunkyo 113-0032, Tokyo, Japan; ytokada@iqb.u-tokyo.ac.jp; 2 Fukushima Medical Center for Children and Women, Fukushima Medical University, Hikarigaoka 960-1295, Fukushima, Japan; kuroda-y@fmu.ac.jp

**Keywords:** human sperm, single-cell pulsed-field gel electrophoresis, DNA fragmentation, reactive oxygen species, dye exclusion test, multi-modal analysis

## Abstract

In our previous studies, OptiPrep and Percoll density gradients separated human motile sperm without DNA fragmentation from immotile sperm with DNA damage. Even in normospermia, over half of the sperm were already immotile, and angle-modulated two-dimensional single-cell pulsed-field gel electrophoresis showed that these were at the end stage of fragmentation. We developed sperm-specific dye- and lectin-exclusion assays to evaluate plasma and acrosomal membranes, mitochondrial endogenous reactive oxygen species, and vacuole negative staining. Comprehensive analyses suggested that they corresponded to sperm that had not yet undergone apoptosis and to those that had undergone apoptotic denaturation. In ICSI, injectable motile sperm that fully meet criteria have an oval-shaped head, intact membranes on both the head and tail, and normal oxidative phosphorylation in cylindrical mitochondria, and they lack vacuoles and DNA damage. Conversely, sperm exhibiting apoptotic signs, such as immotility, plasma membrane damage, and DNA fragmentation, are not injectable. We must establish threshold criteria for injectable sperm; multiple impairments in sperm hinder the study of these issues. The topic of functional impairments in human sperm is too extensive to cover in a single review; for the full scope of the issue, technical guidance for DNA fragmentation analyses is presented in our previous review.

## 1. Introduction

Disorders of spermatogenesis lead not only to decreased sperm production but also to morphological and functional impairments in sperm. Apoptosis regulates spermatogenesis in the testis at the stages of spermatogonia, spermatocytes, and spermatids [1]. More than half of the differentiating spermatogenic cells undergo apoptosis before maturing into sperm and are selectively phagocytosed by Sertoli cells [2].

As described in our previous reports [3,4,5,6,7,8], a combination of centrifugation methods, including sedimentation equilibrium in a discontinuous OptiPrep (OP) density gradient followed by differential velocity sedimentation in a Percoll density gradient, efficiently separates motile sperm without DNA fragmentation from immotile sperm with end-stage fragmentation. This method was originally developed to prepare negative and positive standards for DNA fragmentation analysis [3,4,5,6,7,8].

We have developed several preoperative clinical examination methods to evaluate human sperm morphology and function. These include one-dimensional single-cell pulsed-field gel electrophoresis (1D-SCPFGE) [9,10,11] and angle-modulated two-dimensional single-cell pulsed-field gel electrophoresis (2D-SCPFGE) for detecting DNA fragmentation [7,8]; sperm-specific dye- and lectin-exclusion assays to assess the plasma and acrosomal membranes [6]; the dye-retention assay to evaluate midpiece morphology [6]; assessment of endogenous reactive oxygen species (ROS) in mitochondria [6]; translucent negative staining of internal vacuoles [12,13,14]; indirect immunofluorescent staining for anti-sperm antibodies [5]; and differential staining of the head and tail. DNA fragmentation analyses using 1D- and 2D-SCPFGE are technically complex; guidance to prevent false positives and false negatives is provided in reference [11].

We applied this separation technique to study male infertility, expecting positive results, but encountered technical limitations. The immotile sperm at the final stage were effectively excluded, while motile sperm from earlier stages contaminated the final preparation. During intraoperative sperm pickup for ICSI, medical technologists can only assess motility and gross morphology. The methods mentioned above showed that even after separating motile sperm, there are often multiple potential impairments beyond fragmentation, and the types vary among individuals.

To date, much of the literature has discussed the etiological relationship between a single impairment and male infertility, while multiple impairments in sperm hinder the study of these issues. Based on these observations, multi-modal single-sperm analyses, which integrate multiple pieces of information, are useful for establishing more detailed threshold criteria for injectable sperm.

## 2. The Proportion of Immotile Sperm Undergoing End-Stage Fragmentation in Human Semen Is Much Higher than We Anticipated

Motile and immotile sperm were separated for use as standards in DNA fragmentation analysis; detailed procedures are described in our previous reports [3,4,5,6,7,8].

Video images are included in the Supplementary File. In sedimentation equilibrium in OP (apparent density: 1.085 and 1.17 g/mL; Axis Shield, San Jose, CA, USA), the progressively motile sperm are recovered at the interface layer of OP (Video S1), although the percentage of motility varies among individuals. Their apparent densities are estimated to be less than 1.17 g/mL [5]. Sperm recovered in the OP sediment are almost immotile and highly auto-agglutinated (Video S2), with an estimated density exceeding 1.17 g/mL [5].

**Figure 1:**
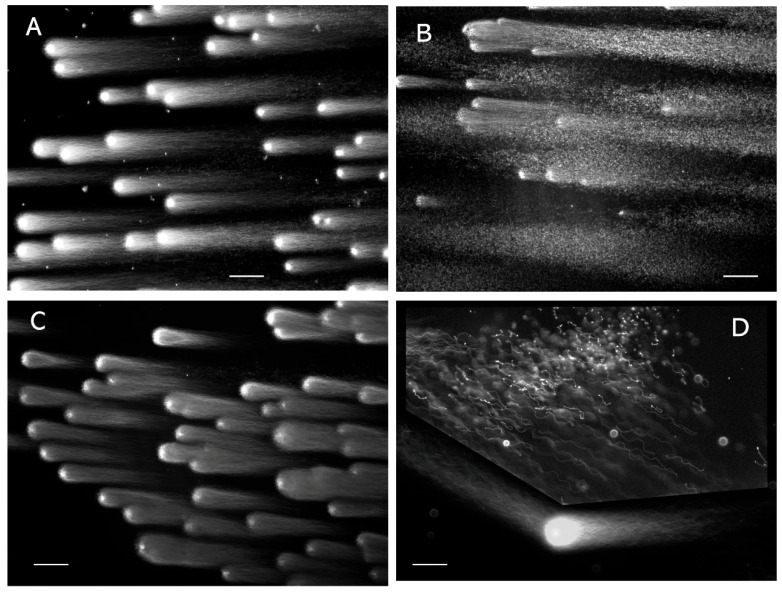
Mono-dimensional single-cell pulsed-field gel electrophoresis and angle-modulated two-dimensional single-cell pulsed-field gel electrophoresis. The details of 1D- and 2D-SCPFGE have been reported previously [7,8,9,10,11]. (**A**–**C**) show the electrophoretic profiles of 1D-SCPFGE. (**A**) The interface layer of OP. (**B**) The sediment of OP. (**C**) The interface layer of OP was further centrifuged using a Percoll density gradient. The sperm were recovered from the sediment. (**D**) A typical profile of 2D-SCPFGE. Scale bars represent 50 µm.

Figure 1 compares the DNA profiles of these fractions using 1D-SCPFGE [9,10,11]. Sperm recovered in the OP interface layer (Video S1) exhibited elongated long-chain fibers with or without fibrous segments (Figure 1A), whereas those in the end stage with granular segments remained contaminated. Conversely, nearly all sperm in the OP sediment (Video S1) discharged uniformly granular DNA segments (Figure 1B). These fractions were collected separately and further purified using a Percoll density gradient (apparent density: 1.12 g/mL; GE Healthcare, Chicago, IL, USA). Motile sperm were recovered in Percoll sediment, where they strongly adhered to the glass surface at the equatorial segment, with the head and tail beating vigorously (Video S3). Adding a small amount of seminal plasma to the suspension or coating on the glass slide released this adhesion, restoring motility (Video S4). The mechanism of this adsorption is discussed later. Sperm in this fraction exhibited a bundle of long-chain fibers from the origin; some discharged a few fibrous segments beyond the tips, and there was no contamination by sperm containing granular DNA segments (Figure 1C).

The specimen shown in the video images and Figure 1 was of high quality rather than moderate quality. The unseparated semen was heterogeneous in motility and DNA fragmentation. 1D-SCPFGE showed that initially, a small number of long fibrous segments appeared beyond the tips of the elongated fibers. As fragmentation progresses, the number of fibrous segments increases while their lengths shorten, and complete DNA degrades into granular segments at end-stage fragmentation [10,11,12,13,14]. Overall, we refer to immotile sperm at end-stage fragmentation as “denatured sperm (DS)” and to motile sperm with a low rate of DNA fragmentation as “purified sperm (PS)”. Among PS preparations, those with a negative DNA fragmentation rate greater than 90% were selected as the negative standard. As described later, DS and PS correspond to those that had not yet undergone apoptosis and those that had already undergone apoptosis and denaturation, respectively. Separation may be caused by increased apparent density due to apoptotic volume decrease [15] and by changes in the hydrodynamic properties of damaged plasma membranes.

The sperm count in Video S2 was noticeably higher than that in Video S1. When examining normospermic semen (66 ± 12 × 10^6^ sperm/mL, 61 ± 10% motility, *n* = 10), an aliquot (1.0 mL) of liquefied human semen was placed on 0.5 mL of isotonic OP and centrifuged at 13,000× *g* for 10 min. The interface and the sediment were collected separately and returned to their original volumes. Microscopic observation overestimated the motility of unseparated semen. The sperm concentrations at the interface were 37 ± 6.6 × 10^6^ sperm/mL and 62 ± 6.4 × 10^6^ sperm/mL in the sediment, respectively. To date, the sperm concentration in human semen has been the primary method for assessing semen quality. However, denatured sperm are unnecessary for evaluating fertility. The number of sperm at the interface layer provides a more accurate measure of semen quality. We recommend analyzing this fraction rather than relying solely on semen microscopy.

Although the sperm in the semen and the OP interfacial layer were motile while sandwiched between the vitreous slide and the cover slip, the head of PS adhered to the glass surface. Adsorption of organic molecules onto silica surfaces is driven primarily by ionic interactions with silanol groups and by hydrophobic interactions with siloxane groups [16]. Adding a small amount of seminal plasma to the suspension released PS from the glass surface. This suggests that some components of seminal plasma selectively adsorb to silanol and siloxane residues, thereby preventing sperm adhesion. Diluting seminal plasma tenfold with pure water is suitable for daily use. A computer-assisted sperm motility analyzer is essential for measuring motion parameters, such as swimming speed. The interaction between the glass and the head significantly influences these parameters. As seminal plasma was removed during purification, the speed decreased. Ultimately, the head became fixed on a slide.

## 3. Sperm-Specific Two-Step Dye and Lectin Exclusion Assays to Observe the Plasma and Organelle Membranes

### 3.1. Observation of the Plasma Membrane on the Head with Reactive Red 195/Reactive Blue 222

As is well known, mammalian sperm differ significantly from somatic cells in membrane organization. Their surface is divided into at least four regions: the acrosomal cap at the front of the head, the posterior part of the head, the midpiece, and the principal and terminal parts of the tail. The acrosomal cap consists of plasma and the outer and inner acrosomal membranes [17,18]. The posterior region of the head is covered only by the plasma membrane; the absence of cytoplasm places the nucleus beneath the membrane.

We developed a sperm-specific, two-step dye-exclusion assay to assess the head plasma membrane [6]. The isotonic reactive red 195 (RR195), a dye that binds to protamine, is incubated with the sperm suspension. The sperm are then fixed and counterstained with reactive blue 222 (RB222). Sperm that allow RR195 to permeate are stained red, indicating damaged membranes.

**Figure 2:**
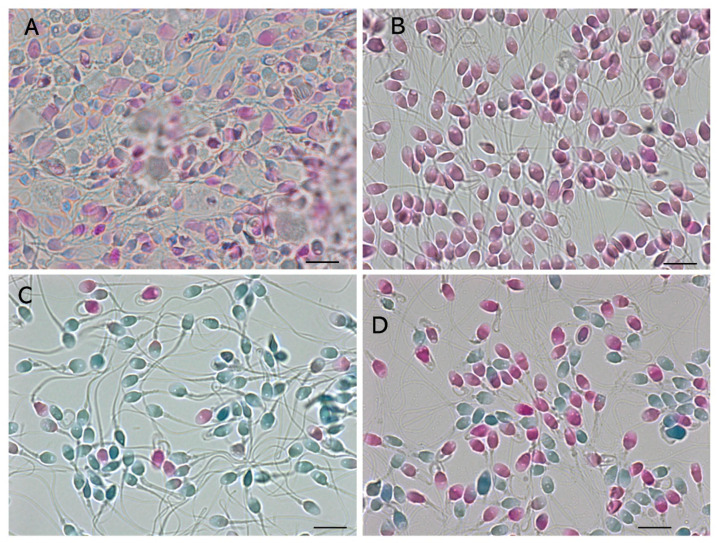
Sperm-specific two-step dye exclusion assay and dye retention assay. The detailed procedures for these assays are described in our previous reports [6,7]. (**A**) Unseparated semen; (**B**) DS. (**C**,**D**) PS. Scale bars are 10 µm.

Normospermic semen contains debris, and more than half of the sperm have damaged plasma membranes (Figure 2A). Processing with OP/Percoll density gradient removed the debris; all heads in DS appeared red (Figure 2B). The sperm in Figure 2C,D correspond to PS and show over 90% motility, while the extent of damage to the head plasma membrane varies between them.

The negative rate of DNA fragmentation measured by 1D-SCPFGE is usually lower than the motility percentage. We propose the working hypothesis that a key factor in the apparent increase in fragmentation is the presence of sperm with damaged head plasma membranes that remain motile. The swim-up method cannot remove this contaminant. Immotility indicates damage to the tail membrane, but motility does not ensure membrane integrity at the head. If the head membrane is damaged, DNA integrity may also be compromised. From a clinical ICSI standpoint, it is crucial to determine whether head membrane damage causes DNA fragmentation. This is why we referred to “threshold criteria for injectable sperm” and “multi-modal analyses” in the preface.

### 3.2. Observation of the Acrosomal Membrane with Cy3- and Alexa-488-Labeled Concanavalin A

**Figure 3:**
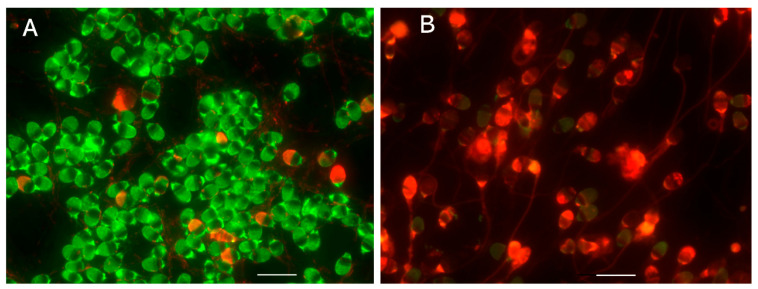
Sperm-specific two-step concanavalin-A labeling. (**A**) PS. (**B**) DS. The scale bars represent 10 µm.

As with RR195/RB222, the acrosomal feature was initially measured using a two-step lectin-exclusion test with Cy3- and Alexa488-concanavalin A (ConA) [6]. In the merged image, red fluorescence indicates that gaps between the plasma and outer acrosomal membranes allowed Cy3-ConA to permeate and bind to the inner acrosomal membrane. Green fluorescence showed that Alexa488-ConA bound to the inner acrosomal membrane after methanol treatment, indicating membrane integrity. Figure 3A,B show sperm corresponding to PS and DS, respectively. The results in the video images and Figure 2 and Figure 3 summarize the status of DS, with the plasma membranes at the anterior and posterior regions of the head and tail being denatured. In contrast to the plasma membrane (Figure 2), the acrosome in PS generally exhibited green fluorescence. The fluctuation range shows less variability among specimens and is of low priority in clinical examination.

## 4. Morphology of the Midpiece and Generation of Reactive Oxygen Species in the Mitochondria

**Figure 4:**
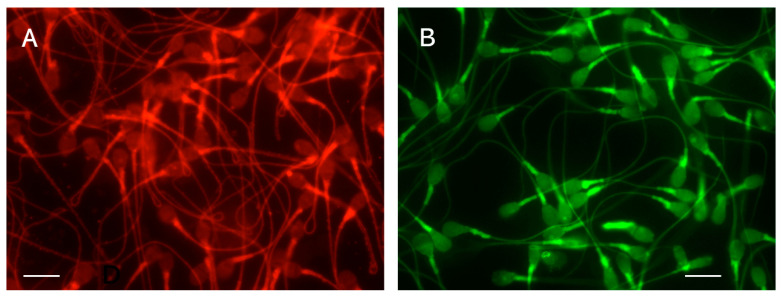
ROS generation in mitochondria and morphology of the midpiece visualized with Mito Tracker FM. Both photographs show the profiles of PS. (**A**) Red fluorescence of CellROX Orange. (**B**) Green fluorescence of Mito Tracker FM. The scale bars represent 10 µm.

Oxidative phosphorylation in the mitochondria is the primary source of reactive oxygen species (ROS), accounting for about 90% of cellular ROS production [19]. Superoxide anions are the most common mitochondrial ROS [19] and are primarily produced in the electron transport chain during oxidative phosphorylation; they are ultimately converted to H_2_O [20]. CellROX Orange [21] (Thermo Fisher Scientific, Waltham, MA, USA) penetrates the mitochondria and reacts with ROS, producing orange fluorescence. Figure 4A shows the PS profile; the midpiece appears uniformly fluorescent. In contrast, no fluorescence is observed in DS. Detecting ROS production in PS is a low-priority functional test, but the photograph provides additional evidence that a sperm remains alive until fixation (Section 9).

The morphology of the midpiece was visualized using Mito Tracker FM [22] (Thermo Fisher Scientific, Waltham, MA, USA). Unlike CellROX Orange, it permeates and remains in the mitochondria regardless of the organelle’s membrane potential (Figure 4B). For example, normal midpieces appeared cylindrical; however, in PS, part of the midpiece deformed, including a knobby bulge. This was used to assess organelle morphology.

## 5. Indirect Immunofluorescent Staining for Anti-Sperm Antibody

**Figure 5:**
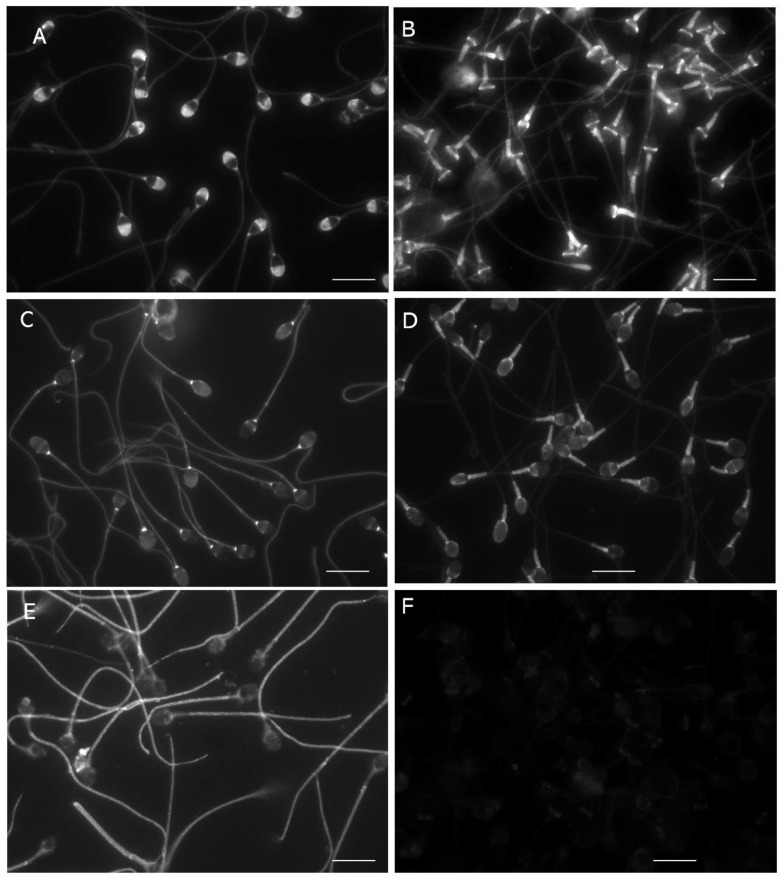
Observation of antigenic sites on PS and DS by indirect immunofluorescent staining and agglutination of PS by anti-sperm antibody. IgG from the sera of the wife and husband was partially purified by ion-exchange absorption on DEAE Sephadex A50. It was made isotonic with 10× concentrated Hank’s solution, then reacted with sperm corresponding to PS and DS. The fluorescent profiles in (**A**–**E**) show the antigenic sites on PS. To improve the CCD device’s sensitivity, the photographs were taken in monochrome. (**A**) The acrosome cap; (**B**) the equatorial segment; (**C**) a point-like organelle at the junction of the head and midpiece; (**D**) the midpiece and (**E**) the principal piece of the tail; (**F**) the profile of DS, reacted with IgG, as shown in (**B**). The scale bars represent 10 µm.

To date, sperm immobilization tests [23], sperm agglutination tests [24], immunobead tests [25], and mixed anti-globulin reaction tests [26] have been used to detect anti-sperm antibodies (ASAs). Immunoglobulin G (IgG) from the sera of the wife and husband was partially purified by ion-exchange absorption on DEAE Sephadex A50 [27]. It was then reacted with sperm fractions corresponding to PS and DS, prepared from the husband’s semen, to assess the clinical significance of ASAs. The localization of antigenic sites on the sperm was assessed by indirect immunofluorescence staining (IIFS) using secondary Alexa 488-conjugated goat anti-human IgG. PS is highly immunogenic for ASAs; both allo- and auto-ASAs were often produced against the acrosome cap (Figure 5A), the equatorial segment and the midpiece (Figure 5B), a point-like organelle at the junction of the head and midpiece (Figure 5C), the midpiece (Figure 5D), and the principal piece of the tail (Figure 5E). DS lost its immunogenicity; Figure 5F shows the profile of DS. As shown in Figure 2B and Figure 3B, membrane-bound antigens may be lost during apoptotic denaturation of the plasma membrane [28]. In addition to these phenomena, our comprehensive observations identified immotility, DNA fragmentation, increased apparent density, acrosomal denaturation, and loss of oxidative phosphorylation during apoptotic denaturation. Since DS consistently outnumbers PS in unseparated semen (Figure 1), ASA testing with DS as the test sample yields false negatives. To date, the sperm agglutination test has been used as the primary test for ASAs [24]. As shown in Figure 1B, DS agglutinates non-immunologically; we often observe masses of still-agglutinated DS in semen. Video S5 shows agglutinated clusters of swimming PS, which move randomly due to their tail beating. To avoid false positives and negatives, the agglutination test and IIFS should be performed on PS prepared from the husband.

Unlike monoclonal IgG, serum often contains heterogeneous polyclonal IgGs that bind at multiple sites on sperm. In our previous report, 23 of 67 IgG fractions from women in infertile couples exhibited at least one antigenic site. Of 36 positive sites, the most common were the equatorial segment (seven sites), the principal piece of the tail (five sites), the junction of the head/midpiece (four sites), and the midpiece (three sites). We also observed 10 women who became pregnant spontaneously, 4 of whom were positive for ASAs in sera submitted for pregnancy testing [5]. This finding suggests an important point: although IIFS visualizes the antigenic site, many molecules are present there, and binding of ASAs to the husband’s PS alone cannot diagnose immune infertility. Swimming sperm from men in infertile couples often have various impairments. To discuss the pathological significance of immune infertility, we must exclude other potential causes of infertility. Making this issue clear requires multi-modal single-sperm analyses, which we discuss in Section 9.

## 6. Morphologies of the Organelles in Human Sperm

**Figure 6:**
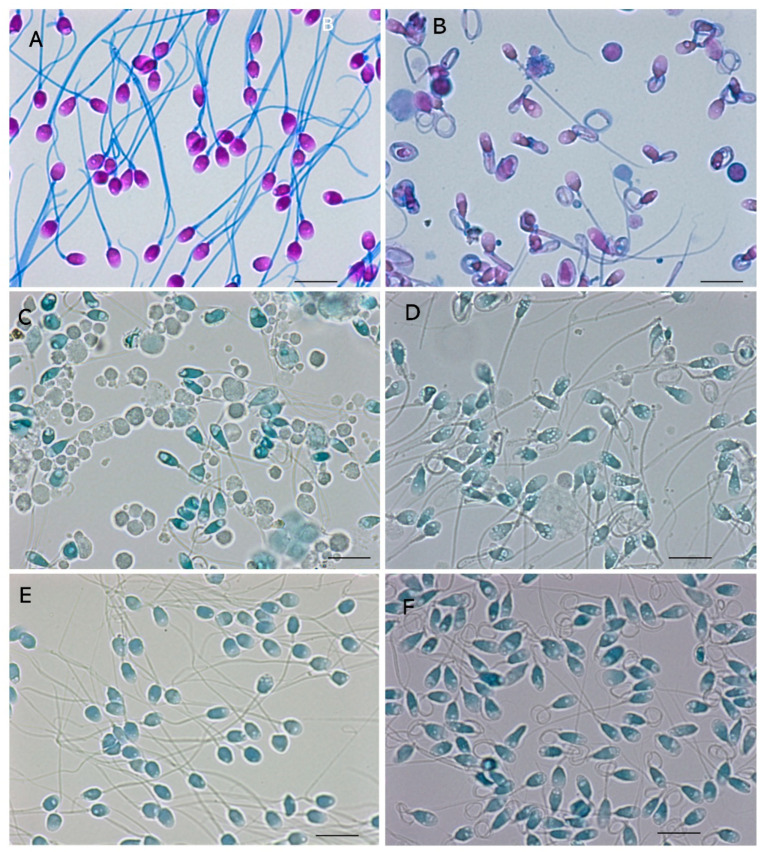
Morphology assessments of the head, tail, and vacuoles. (**A**) Reactive red 250 (0.001%, 0.1 mol/L NaHCO_3_-Na_2_CO_3_, pH 10 for 5 min) selectively stained the head of PS red, and Coomassie Brilliant Blue G-250 (0.01%, 0.1 mol/L acetate buffer, pH 4.7) stained the tail blue. (**B**) The sperm with tail dysplasia were stained in a similar manner to (**A**). (**C**,**D**) RB5 staining of the unseparated semen. (**E**,**F**) RB5 staining of the purified sperm corresponding to PS. Scale bars represent 10 µm.

Figure 6 summarizes the subcellular morphologies of sperm in semen, PS, and DS. The strict criteria established by Kruger et al. [29] focus on the head outline. Differential staining with reactive red 250 and Coomassie brilliant blue G-250 highlights the head and tail. The midpiece and acrosome were visualized using Mito Tracker FM (Figure 4B) and concanavalin A (Figure 3A). Together, these stains reveal the entire sperm. Figure 6A,B show the profiles of PS and sperm with tail dysplasia. All the figures in this review emphasize the importance of comprehensive observation of the morphology and function of the organelles to understand the entire sperm.

Human sperm often contain vacuoles; we initially visualized them using the translucent stain Reactive Blue 2 (RB2) [12]. Under normal bright-field optics, the heads appeared as translucent, bluish bodies, with internal vacuoles visible as toneless spots [12,13]. In our recent report, we screened 12 commercially available reactive dyes, and Reactive Black 5 (RB5) provided higher contrast than RB2. Subsequently, 30 RB5 analogs were synthesized, and structure–activity relationship analyses identified compound 2221 as the best dye for visualizing vacuoles under normal bright-field optics [14]. RB5, which contains four ionizable sulfonates, stained leukocytes and sperm at neutral pH but selectively stained sperm at pH 10. The sulfonate groups electrostatically bind to guanidyl residues in Arg-rich domains of protamines, but not to amino residues in lysine (Lys). The pH-dependent cellular specificity arose from differences in Arg content between protamines and histones [30]. As shown in Figure 6C, RB5 selectively stained sperm in unseparated semen but did not stain debris.

To date, much of the literature has discussed the etiological relationship between vacuoles and DNA fragmentation, yet no consensus has been reached [31,32]. In general, as semen quality declines, the head outline deforms, and the size and number of vacuoles increase, though their features vary significantly among individuals. A semen sample in Figure 6D contains small sporadic vacuoles in the deformed head. As discussed later in Section 10, we performed digital morphometric analysis to establish the “referential oval” [14]. As shown in Figure 6E,F, the current process for separating motile sperm cannot exclude those with vacuoles. The area, head aspect ratio, and vacuole profiles are heterogeneous across sperm samples. When comparing oval and amorphous heads, we empirically understand that the former is normal. As noted in the preface, our main goal is to determine the threshold criteria; it is often difficult to define where normal ends and impairment begins. Several PS preparations used as negative standards contained various vacuoles; currently, we believe vacuoles may not be directly responsible for DNA fragmentation, but we cannot exclude the possibility of pathological vacuoles. Clarifying this issue requires multi-modal single-sperm analyses, which we discuss in Section 9.

## 7. Lessons Learned from the Hypo-Osmotic Swelling Test

Extracellular hypo-osmotic pressure damages plasma and organelle membranes. The fibrous sheath is a cytoskeletal structure surrounding the axoneme and outer dense fibers in the main part of the sperm tail. The hypo-osmotic swelling (HOS) test assesses swelling of the terminal piece without coverage to predict the integrity of the human sperm plasma membrane [33]. Our previous report revalidated the HOS test principle using the RR195/RB222 assay [6]. In PS, the 78% exclusion rate for RR195 was used for the examination. When osmotic pressure was halved, all sperm became immobile, and the terminal piece coiled. Meanwhile, the rate decreased to 64%, and the fluorescent intensities of both dye retention (Mito Tracker FM) and ROS generation (CellROX Orange) decreased from levels in the isotonic medium. Mitochondrial functions did not degenerate completely. The plasma membrane of the head and the organelle membrane within the mitochondria were more tolerant of hypo-osmotic pressure than those on the terminal piece of the tail. We are concerned that the posterior region of the head is surrounded solely by the plasma membrane, and that its close attachment to the nucleus may enhance its tolerance.

The HOS test results indicate a localized change in the terminal part of the tail that profiles cannot capture across the entire sperm membrane. Some studies have claimed that it is very useful for selecting viable sperm for ICSI, especially when using non-motile or testicular sperm [34,35]. However, they extend this idea too far, as we emphasized in this review: apoptosis regulates spermatogenesis in the testis [1], and nearly all immotile sperm in semen have already undergone fragmentation (Figure 1B and Figure 2B). This situation highlights the critical need to gather comprehensive information and integrate the data.

## 8. Single-Nuclear DNA Fragmentation Analyses

### 8.1. How to Prevent Artifactual False Positives and Negatives in DNA Fragmentation Analyses

The topic of functional impairments in human sperm is too extensive to cover in a single review article; therefore, we split it into two reviews. The discussion of DNA fragmentation analyses is presented in reference [11]. To provide the full scope of the issue in this review, a summary is presented in this section.

Over the past two decades, nonspecific single-nuclear DNA fragmentation in human sperm has drawn the most attention in ART, and the comet assay [36,37], sperm chromatin structure assay [38,39], sperm chromatin dispersion test [40,41], and terminal deoxynucleotidyl transferase-mediated dUTP nick-end labeling assay [42] have been considered gold standards. We developed single-cell pulsed-field gel electrophoresis techniques, including one-dimensional (1D-SCPFGE) and angle-modulated two-dimensional (2D-SCPFGE), to detect naturally occurring DNA fragmentation at moderate to early stages [3,4,7,8,9,10,11]. Revalidation using comparative standards such as PS and DS showed that the traditional methods failed the initial qualitative validation [3,4,7,8,9,10,11].

The term “stage” is defined by the electrophoretic profile: “early stage” is defined as fewer than several long fibrous segments observed at the inner angle of the elongated fibers in 2D-SCPFGE; “end stage” is defined as almost all DNA shredded into granular segments. At present, “moderate” denotes an intermediate stage qualitatively, and it lacks an exact numerical definition.

To illustrate, we briefly describe cumulative technical failures in the comet assay. The assay evaluates DNA damage by counting granular segments, known as the comet tail [36,37]. These segments originate from DS (Video S2 and Figure 1B). Conventional agarose gel electrophoresis can move granular segments, but SCPFGE is essential for elongating DNA fibers [11]. Double-strand breaks (DSBs) are the most challenging DNA lesions to repair, and the critical number of DSBs in a nucleus can be very low [43,44]. Exceeding this threshold can lead to fertilization failure or pregnancy loss. Some of the literature has discussed the effects of DNA damage on fertilization, post-implantation embryo development [45], and sperm-derived congenital anomalies in ART [46]. The aim of DNA fragmentation analysis in clinical assisted reproductive technology (ART) is to detect early signs of DNA fragmentation in PS; the criteria for test specimen eligibility should be strictly defined. The technical platform used in the comet assay does not meet the required sensitivity.

To ensure the sensitivity and quantitative accuracy of SCPFGE, we need to standardize measurement principles, comparative standards, the calibration curve, required sensitivity, and eligibility criteria for test sperm. Currently, 2D-SCPFGE with angle rotation is the most sensitive imaging method for single-nuclear DNA fibers (Figure 1D). Naked chromosomal DNA fibers are highly fragile and susceptible to mechanical and chemical damage; a detailed work procedure manual is essential to prevent artifactual false positives and negatives. Due to space limitations, please refer to our recent reports and reviews [7,8,9,11].

### 8.2. Environmental Impact on DNA Integrity—Seminal Oxidative Stress and ROS-Induced Automatic Intoxication

Much of the literature has focused on the impact of oxidative stress on DNA integrity [47]. Some studies have examined the effects of ROS on DNA fragmentation [48]; almost all oxidants can damage DNA. To address this issue, we must consider certain environmental pollutants as potential confounding factors; for instance, the recent literature reports that per- and polyfluoroalkyl substances contribute to male infertility through oxidative damage [49,50].

Our previous report [51] examined how hydroxyl radicals cause DNA cleavage; using 1D-SCPFGE, it was observed that DNA in membrane-excluded PS was cleaved by the prooxidant action of ascorbic acid in a dose-dependent manner. In contrast, swimming sperm surrounded by intact plasma membranes were protected from extracellular ROS [51]. The key point is that this quantitative result is based on a limited experimental design. When using unseparated semen as the experimental material, the plasma membrane acts as a shield against extracellular ROS, which are scavenged by antioxidants [52] and by antioxidative enzymes such as superoxide dismutase [53] present in the seminal plasma. Furthermore, most sperm are at the end stage of fragmentation (Video S2 and Figure 1B), making it difficult to determine whether the DNA cleavages are newly generated by ROS. As mentioned in Section 8.1 on experimental conditions, criteria for selecting test samples should be rigorously defined to ensure accurate testing.

From another perspective, we should focus on ROS-induced autointoxication of swimming sperm rather than on the effects of seminal oxidative stress. During the preparation of motile sperm from semen, seminal plasma is replaced with an artificial culture medium that typically contains lactic or pyruvic acid for in vitro fertilization. This replacement results in the loss of antioxidant protection [52] and the antioxidant enzyme system [53]. While oxygen is necessary as the final electron acceptor in the electron transport chain for ATP production, the presence of lactic or pyruvic acid in an atmospheric environment or in 5% CO_2_-air overpromotes oxidative phosphorylation, leading to excessive ROS production, cellular damage, and toxicity [54]. We observed daily that PS cultured in CO_2_-air for 1 day were immobilized, whereas those cultured in 2% O_2_-93% N_2_-5% CO_2_ maintained motility. From a prophylactic perspective, we recommend holding PS in a hypoxic environment and/or adding appropriate sperm-permeable active oxygen scavengers.

To establish negative standards for fragmentation analysis, we first prepared several PS specimens and then examined the motility and integrity of their head plasma membranes. The selected candidates underwent certification testing using 2D-SCPFGE. We confirmed that the intracellular shield separating the nucleus from the midpiece protects DNA integrity in swimming PS for up to 1 h. This value is based on our work manual for preserving the sequential integrity of negative standards [7,8]. We replaced the candidates with a non-aqueous, anti-freezing organic medium containing a ROS scavenger and EDTA within 1 h, without waiting for the test result. If PS is cryopreserved without this protectant, ice crystals mechanically cleave the sequence, and placing PS in an organic solvent eliminates dissolved oxygen.

## 9. Prospects for Multi-Modal Single-Sperm Assessment

In previous sections, we focused on purifying motile sperm and assessing their morphology and function using single-modal analyses. As noted earlier, OptiPrep and Percoll density gradients effectively remove DS, but early-stage, still-motile sperm can contaminate the final preparation. As semen quality declines, contamination by immotile DS also increases. 1D- and 2D-SCPFGE and phase-contrast microscopy readily distinguish them; however, once fixed, stained, and lysed for other single-modal analyses, their differences often become unclear. From this review’s perspective, the injectable motile sperm that fully meet the criteria have an oval-shaped head, intact membranes on both the head and tail, normal oxidative phosphorylation in cylindrical mitochondria, and no vacuoles or DNA damage. We understand that a sperm showing signs of apoptosis, such as immotility, membrane damage, and DNA fragmentation, is not suitable for injection, and it is essential to establish injectable thresholds for other impairments. Clinically, it is urgent to determine whether a sperm with a damaged head plasma membrane, yet still motile, responds to DNA fragmentation (Section 3.1) and whether pathological vacuoles truly exist (Section 6). The immediate goal is to incorporate assessments of plasma membrane damage and oxidative phosphorylation, a surrogate marker of motility, into the other analytical methods.

**Figure 7:**
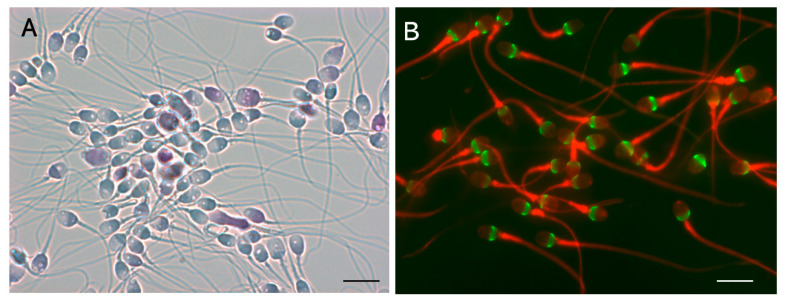
Dual-modal single-sperm analyses. (**A**) RR195/RB5 staining for simultaneous observation of the integrity of the head plasma membrane and the internal vacuoles; (**B**) simultaneous observation of antigenic site and ROS generation in the mitochondria. Scale bars represent 10 µm.

After incubating PS with RR195, the counterstain was switched from RB222 to RB5 to simultaneously assess membrane damage and internal vacuoles. Unlike the original single-modal RB5 staining (Figure 6C–F), the head with an intact plasma membrane contained vacuoles (Figure 7A). This specimen contained a high percentage of sperm with undamaged head membranes, along with many internal vacuoles. Our previous observations showed a weak correlation between their incidences. PS was incubated with partially purified IgG, which has been confirmed to bind antigens at the equatorial segment. The mixture was placed on a glass slide and then reacted with Alexa-488-conjugated goat anti-human IgG in the presence of CellROX Orange. The merged image in Figure 7B shows that ASAs bound to the antigenic site on live sperm, producing ROS. As mentioned in Section 5, sperm surface antigens are lost during apoptosis; the orange fluorescence in the midpiece serves as a surrogate marker of live sperm.

From another perspective, RR195 may be used for intraoperative sperm selection in ICSI, in which swimming sperm stained red are excluded from the candidate pool. Because it binds to protamines, reproductive toxicity must be closely monitored. We aim to identify a dye that is completely excluded by the intact plasma membrane; this will require detailed exposure experiments conducted in accordance with reproductive toxicity guidelines before clinical application.

## 10. Paradigm Shift Beyond Semen Observation

In brief, our work has progressed through the following steps: (1) development of procedures to separate human motile sperm; (2) development of molecular biology-based tests to evaluate the separated motile sperm fraction; (3) validation of the principles; (4) statistical univariable analysis for each test; (5) cohort studies using multivariable analyses; (6) defining threshold criteria for “injectable sperm”; (7) reconfirmation of clinical outcomes based on the newly defined criteria. We are now at stages 2 to 4, and we forecast 5 to 10 years of cohort studies before reaching a conclusion. In nature, this review will be published 10 years later; our clinical opinions are not complete products. Table 1 summarizes the evaluation of the examinations introduced in this review. For details of Nos. 1–6, see reference [11].

The main paradigm shift in this review is that observing unseparated semen has limited value for predicting human sperm fecundity. The number of sperm collected at the OP interface layer provides a more precise estimate of semen quality (Section 1 and Section 2). Even after separating the motile sperm, the sample often includes various potential subcellular impairments, with different types and severities among individuals (Section 3, Section 4, Section 5, Section 6, Section 7, Section 8 and Section 9). At present, 6, 7, 8, 13, and 14 in Table 1 are prospective candidates for detecting potential impairments.

Multi-modal single-cell analysis for PS is a future alternative beyond the WHO framework [55]. Some pioneering efforts have already hinted at the potential of future technologies [56,57]. They demonstrated that AI-based morphological analyses evaluate head, midpiece, and tail morphology in real time, enabling classification of more than 11 types of abnormal sperm morphology in unstained samples. Bright-field optics cannot detect all subcellular impairments in unstained sperm. Our recent report highlighted serious issues in the analysis of human sperm head morphology [14]. A CCD with a resolution of at least 4000 × 3000 pixels is necessary to accurately digitize the outline and vacuoles of PS. According to Kruger’s strict criteria [29], a threshold of 4% smooth oval heads in an ejaculate serves as a de facto standard for better fertilization outcomes in clinical ART. As noted in Section 6, the head aspect ratio of PS showed significant variation, and we could not establish a clear numerical reference to classify it as normal or abnormal [14]. A computer-assisted sperm motility analyzer was used to quantify the motion parameters of human sperm. As shown in Section 2, the interaction between PS and the glass surface significantly affected these parameters. Most major problems arise from the lack of established standards and calibration curves to quantify the results. In this review, we highlight the tendency to overestimate results based on low-accuracy technical platforms. The accuracy of multi-modal single-sperm analyses depends on the quality of the original data.

Our approaches open two pathways. The first one highlights the benefits of carefully selecting high-quality sperm, which improves therapeutic outcomes and clinical safety. In daily practice, however, we often encounter the downside: comprehensive evaluation, along with current single-modal analyses, reveals previously unseen issues, indicating that sperm quality is worse than expected. The future paradigm shift aims to establish threshold criteria to interrupt infertility therapy. More detailed information from multi-modal single-sperm analysis will help to establish more precise criteria.

## Figures and Tables

**Table 1:** Summary of clinical examinations discussed.

	Examination	Purpose	Significance in Daily Practice
1	comet assay	DNA fragmentation	insufficient sensitivity
2	sperm chromatin structure assay	DNA fragmentation	error of principle
3	sperm chromatin dispersion test	DNA fragmentation	error of principle
4	TUNEL assay	DNA fragmentation	insufficient sensitivity
5	1D-SCPFGE	DNA fragmentation	chemical and enzymatic cleavage analyses
6	2D-SCPFGE	DNA fragmentation	naturally occurring fragmentation, semiquantitative
7	observation of fractionated sperm	net amount excluding apoptotic sperm	high
8	sperm-specific 2-step dye exclusion assay	head plasma membrane integrity	high
9	sperm-specific 2-step lectin exclusion assay	acrosomal integrity and localization	low
10	ROS generation in mitochondria	TCA cycle activity	surrogate marker of motility on a photograph
11	Mito Tracker FM	midpiece morphology	optional
12	immunofluorescent assay	anti-sperm antibody (ASA) antigen localization	optional
13	reactive red 250-CBB staining	simultaneous staining of head and tail	high
14	RB2 and RB5 staining	vacuoles in the head	high
15	hypo-osmotic swelling test	plasma membrane integrity	error of principle
16	multi-modal single-sperm assessment	ASA and the TCA cycle	future examination not in practice

## Data Availability

The original contributions presented in this study are included in the article/Supplementary Materials. Further inquiries can be directed to the corresponding author.

## Supplementary Materials

The following supporting information can be downloaded at https://www.mdpi.com/article/10.3390/biom16070928/s1: Video S1: Sperm recovered at the interface layer of OP. Video S2: the sperm in the OP sediment. Video S3: The sperm in Video S1 were further separated in a Percoll density gradient. Those recovered in the sediment. Video S4: Adding a small amount of seminal plasma to the sperm in Video S3 or coating the glass slide releases this adhesion, restoring motility. Video S5: Image of agglutinated PS.
